# Effects of embolic agents with different particle sizes on interventional treatment of uterine fibroids

**DOI:** 10.12669/pjms.316.7955

**Published:** 2015

**Authors:** Xigong Wang, Zhengfu Zhang, Jirong Pan, Weizhong Zhang

**Affiliations:** 1 Xigong Wang, Department of Cancer Intervention, Qingdao Central Hospital, Qingdao 266042, China; 2 Zhengfu Zhang, Department of Radiology, Qingdao Central Hospital, Qingdao 266042, China; 3 Jirong Pan, Department of B Ultrasonic Room, Qingdao Municipal Hospital, Qingdao 266071, China; 4 Weizhong Zhang, Department of Interventional Radiology, Qingdao Municipal Hospital, Qingdao 266071, China

**Keywords:** Uterine fibroid, Embolic agent, Particle size, Quality of life, Ovarian function

## Abstract

**Objective:**

To compare the effects of embolic agents with different particle sizes on interventional treatment of uterine fibroids (UFs).

**Methods:**

One-hundred and thirty patients with UFs were divided into a treatment group and a control group (n=65) by random draw. All patients were treated by uterine artery embolization, with the treatment group using 200 μm polyvinyl alcohol (PVA) particles and the control group using 500 μm PVA particles.

**Results:**

The success rate of embolization was 100%. After intervention, the treatment group was significantly less prone to complications such as lower abdominal pain, fever, nausea, vomiting and bleeding than the control group (P<0.05). The follicle-stimulating hormone levels of both groups were similar before and after intervention, and there were also no significant inter-group differences. The uterine and UF volumes of both groups significantly decreased six months after intervention (P<0.05), and those of the treatment group were significantly lower (P<0.05). The two groups had similar physical function, role-physical, bodily pain and general health scores before intervention, but the treatment group scored significantly higher than the control group did six months after intervention (P<0.05).

**Conclusion:**

Interventional embolization can well treat UFs, without apparently affecting ovarian functions. Small-sized PVA particles can improve the quality of life by shrinking the uterus and UFs as well as by reducing the risks of complications.

## INTRODUCTION

Uterine fibroid (UF), which is a benign tumor composed by proliferated uterine smooth muscle tissues and various numbers of fibrous connective tissues, is the most common female pelvic disease threatening women of childbearing age.^1^ Clinically manifested as abnormal uterine bleeding, pelvic pressure, frequent urination, dysuria, urinary retention, pain and loss of fertility, UFs severely endanger female physical and psychological healths.^2^,^3^ The pathogenesis of UFs remains unclear hitherto, which has most been associated with the changes of extracellular matrix, environmental factors, progesterone, growth factors, gene mutation and estrogen as sex hormone-dependent tumors.^4^,^5^ UFs have been traditionally treated by panhysterectomy, myomectomy and hormone therapy, but surgeries negatively affect patients both physically and psychologically. Besides, hormone therapy, which mainly employs progesterone and gonadotropin-releasing hormone, easily leads to recurrence and even osteoporosis.^2^

Recently, minimally invasive uterine artery embolization (UAE) has been widely applied in clinical practice due to minor traumas, high tolerance, scare complications and satisfactory therapeutic effects. In addition, the uterus can be retained for the patients who have reproductive demands. In general, eligible embolic agents only mildly or do not affect normal tissues and organs, and do not result in obvious complications such as pain.^6^ The aim of UAE is to block the blood supply of UFs before capillaries by using microparticles, allowing ischemic necrosis and atrophy.^7^

Being highly stable, biocompatible and non-toxic, polyvinyl alcohol (PVA) particles have permanent embolizing effects in human body and do not enter distal radial arteries or small spiral arteries, thus maintaining myometrial vessels unobstructed.^8^ Nowadays, 500 μm PVA particles are used most often, and 200 μm ones have also been applicable.^9^ In this study, the effects of embolic agents with different particle sizes on interventional treatment of UFs were compared, aiming to provide evidence for future use.

## METHODS

### Subjects

One-hundred and thirty patients with UFs, who were treated in our hospital from July 2011 to February 2014, were selected. All of them were diagnosed by clinical symptoms, gynecological examination and imaging data (B-scan ultrasonography and MRI, etc.).

### Inclusion criteria

Women in the child-bearing period who had given birth, with increased menstruation as the main symptom; clinically manifested as menstrual changes, pelvic pressure and dysmenorrhea; with over 6 months of follow-up data; unwilling to receive surgeries or refused to do so owing to future reproductive demands; with written consent form; diagnoses as submucosal or intramural fibroids by imaging, without other uterine pathological changes.

### Exclusion criteria

With large angiographic contraindications; with pedunculated fibroids and enlarged uteri while being over 25-week pregnant; with severe arteriosclerosis and the elderly. The patients were divided into a treatment group and a control group (n=65) by random method, and the two groups had similar age, disease course, uterine volume, UF volume, UF status and body mass index before intervention (P>0.05) (Table-I).

**Table-I:** Baseline data.

Index	Treatment group (n=65)	Control group (n=65)	χ^2^ or t	P
Age (year)	41.83±4.92	41.78±5.00	0.153	>0.05
Disease course (year)	3.76±1.08	3.72±1.00	0.221	>0.05
Uterine volume (cm^3^)	290.78±14.88	291.73±16.33	0.045	>0.05
UF volume (cm^3^)	114.87±13.00	116.37±12.63	0.183	>0.05
Body mass index (kg/m^2^)	22.38±2.61	22.41±3.09	0.089	>0.05

### Treatment methods

All patients were subjected to UAE and digital subtraction angiography by using GE 64-layer spiral CT scanner and Phillips V-5000 X-ray system. Non-ionic contrast agent iopamidol was used as the contrast agent, and 200 μm and 500 μm PVA particles (Cook) were used as the embolic agents. The treatment group and the control group were treated with 200 μm and 500 μm PVA particles respectively. UAE was conducted within 3-7 days after menstruation. All patients were locally anesthetized by 2% lidocaine, and catheterized through the right femoral artery with the Seldinger technique. Then bilateral internal iliac arteries were horizontally catheterized on the abdominal aortic bifurcation by 5F catheters before DSA, and Cobra catheters were inserted into left and right uterine arteries respectively. Afterwards, DSA was carried out again to display the blood supply characteristics of UFs. Under imaging guidance, PVA particles were thoroughly mixed with contrast agent and slowly injected into UF blood vessels. A total of 40-150 mg embolic agents were used, with the average of 90 mg. After UAE, aortic angiography was performed before removal of the catheters.

### Observation indices

The success rate of UAE was observed, and postoperative complications after intervention, such as abdominal pain, fever, nausea, vomiting and bleeding, were observed. Uterine and UF volumes were measured six months before and after treatment by using color Doppler ultrasound scanner.

Six months before and after intervention, fasting levels of follicle-stimulating hormone (FSH) were measured to monitor the changes of ovarian function.

The overall therapeutic effects were evaluated six months after intervention. Marked effective: Significant reduction of menstrual flow, uterine and UF volumes decreased by ≥50%; effective: significant reduction of menstrual flow, uterine and UF volumes decreased by 20%-50%; ineffective: mild or no reduction of menstrual flow, uterine and UF volumes decreased by <20%.

The quality of life was investigated with SF-36 health survey by telephone or clinical visit six months before and after intervention. The survey involved physical function, role-physical, bodily pain and general health status, and a higher score meant better quality of life.

### Statistical analysis

All data were analyzed by SPSS 17.0. The numerical data were compared by the χ^2^ test of fourfold or row-column tables. The categorical data were expressed as (mean ± standard deviation). Intra- and inter-group comparisons were performed with univariate analysis of variance and t test. P<0.05 was considered statistically significant.

## RESULTS

### Complications

The success rate of UAE was 100%. After intervention, the treatment group was significantly less prone to complications such as lower abdominal pain, fever, nausea, vomiting and bleeding than the control group (P<0.05) (Table-II). All complications were relieved by further treatment, without affecting normal menstrual cycle.

**Table-II:** Complications after intervention (n).

Group	Case number (n)	Lower abdominal pain	Fever	Nausea and vomiting	Bleeding	Total
Treatment group	65	2	2	2	1	7 (10.8%)
Control group	65	4	4	5	4	17 (26.2%)
χ^2^						8.553
P						<0.05

### Changes of uterine and UF volumes

The uterine and UF volumes of both groups significantly decreased six months after intervention (P<0.05), and those of the treatment group were significantly lower (P<0.05) (Table-III).

**Table-III:** Uterine and UF volumes before and after intervention (cm^3^, x±s).

Group	Case number (n)	Uterine volume		UF volume	
		Before	Six months before	Before	Six months before
Treatment group	65	290.78±14.88	168.24±12.87*	114.87±13.00	23.94±9.44*
Control group	65	291.73±16.33	123.76±13.99*	116.37±12.63	44.73±10.67*
t		0.045	14.998	0.183	19.334
P		>0.05	<0.05	>0.05	<0.05

* Compared with the data before intervention,P<0.05.

### FSH level changes

The FSH levels of both groups were similar before and after intervention, and there were also no significant inter-group differences (Table-IV).

**Table-IV:** FSH level changes before and after intervention (IU/L, x±s).

Group	Case number (n)	Before	Six months before	t	P
Treatment group	65	9.99±4.38	10.09±4.33	0.453	>0.05
Control group	65	10.10±3.89	10.66±5.21	0.500	>0.05
t		0.145	0.398		
P		>0.05	>0.05		

### Scores of quality of life

The two groups had similar physical function, role-physical, bodily pain and general health scores before intervention, but the treatment group scored significantly higher than the control group did six months after intervention (P<0.05) (Table-V).

**Table-V:** Scores of quality of life.

Group	Case number (n)	Physical function	Role-physical	Bodily pain	General health status
*Before*					
Treatment group	65	55.38±5.33	48.98±6.37	56.92±5.09	57.11±6.02
Control group	65	55.37±4.02	48.77±6.20	57.00±5.22	57.28±6.22
t		0.008	0.056	0.067	0.055
P		>0.05	>0.05	>0.05	>0.05
*After*					
Treatment group	65	83.47±6.44	84.78±7.03	84.29±6.00	85.92±5.11
Control group	65	74.93±5.09	78.46±6.00	75.99±5.30	71.63±6.31
t		6.983	4.983	6.449	8.444
P		<0.05	<0.05	<0.05	<0.05

## DISCUSSION

As an essential organ of female, the uterus plays a crucial role in formation of menstruation, giving birth, endocrine function, pelvic floor support, ovarian blood supply and hemodynamic maintenance^10^ by producing a variety of bioactive substances. Meanwhile, modern women have paid particular attention to the improvement of quality of life, which allows wide application of novel minimally invasive treatment methods that can retain the uterus.^11^ UFs are benign tumors comprising proliferated uterine smooth-muscle tissues and fibrous connective tissues. Without apparent symptoms in most cases, UFs are usually accidentally detected during pelvic examination. As UFs progress, the patients begin to suffer from menstrual change, lower abdominal mass, pelvic pressure, increased vaginal discharge, abdominal pain, infertility and secondary anemia, etc. The pathogenesis of UFs remains unclear, which is commonly attributed to hormone dependence because they are mainly found in women with ovarian hyperfunction.^12^

Traditionally, UFs are treated with panhysterectomy to prevent possible relapse and malignant transformation. However, this method is greatly traumatic and destroys the integrity of the pelvic floor, thus probably decreasing the quality of life after intervention and negatively affecting patients psychologically as well.^13^ Although subtotal hysterectomy has lower risk and reserves the cervix, malignant transformation may occur. Besides, conservative therapy with hormones requires long-term administration and easily leads to complications. In the meantime, the risk of malignant transformation is raised after stopping drug administration owing to endocrine disorders and rapid growth of UFs.^14^

Anatomically speaking, blood in a normal uterus is mainly supplied by bilateral uterine arteries and occasionally by ovarian arteries, constituting a rich vascular network. Therefore, embolizing the end of uterine arteries can render UF tissues to undergo ischemic necrosis and atrophy, whereas the normal myometrium that has abundant blood vessels does not undergo massive necrosis, thus maintaining normal uterine tissues intact and physical functions normal.^15^ In the meantime, interventional embolization therapy, which does not require laparotomy, is minimally invasive. Clinical symptoms can be alleviated while retaining the physical functions of the uterus and ovary, and this procedure can be repeated upon incomplete treatment or relapse. Moreover, the highly biocompatible, nontoxic PVA particles remain permanently effective in human body.^16^ PVA particles, when used, do not enter distal radial arteries or small spiral arteries, which conforms to the vascular anatomical requirements. In this study, the success rate of UAE was as high as 100%.

Since the diameters of small uterine arteries range from 300 to 1000 μm and those of small arteries dominating the basal layer of the endometrium are from 30 to 300 μm, PVA particles diametered less than 300 μm are commonly selected. In comparison, 200 μm PVA particles can enter deep uterine vessels, thus allowing more effective UAE that evidently shrinks the uterus and UFs as well as improves the prognosis.^17^ The uterus and UF volumes of both groups significantly shrank six months after intervention (P<0.05), and those of the treatment group changed significantly more obviously (P<0.05). Though UAE barely induces postoperative complications, lower abdominal pain, fever, nausea, vomiting and bleeding should be treated in time. Moreover, 500 μm particles may lead to more complications because small uterine arteries were diametered less than this and UFs cannot be completely embolized. After intervention, the treatment group had significantly less complications such as lower abdominal pain, fever, nausea, vomiting and bleeding than the control group did (P<0.05). All complications were mitigated by further treatment, without affecting normal menstrual cycle.

By using 200 µm PVA particles to embolize uterine arteries, Hehenkamp et al.^18^ found that the minimum arterial diameter for these particles to remain was 300 µm. As the smallest particles used hitherto, they successfully blocked the vascular network surrounding fibroids for a long time, without affecting normal uterine supply. Aziz et al.^19^ performed UAE through uterine arteries by using PVA particles diametered 200 µm and hysterectomy thereafter, and found that these particles were mainly distributed in parauterine and uterine wall surface arteries diametered 1-2 mm (minimum: >300 µm). Pelage et al.^20^ embolized UFs by using 500 µm PVA particles, but MRI analysis showed considerable residual blood supply and easy relapse of these UFs. James et al.^21^ reported that UFs in 20% of 200 patients who were treated by 500 µm PVA particles for embolization relapsed after one year of follow-up. Besides, these particles exerted obvious therapeutic effects only on UFs with small volumes and rich blood supply.

UFs depend on hormones, the blood supply of which can be blocked by UAE. In general, hormones cannot enter UF tissues along with blood flow, and once their receptors are destructed, UFs are bound to further shrink due to the resulting local environment alike that of menopause.^22^ Regardless, there remains controversy over the influence of UAE on menstruation and ovarian functions, for which FSH that promotes follicular development is often used as a reliable index for determining ovarian failure.^23^ The FSH levels of both groups did not differ significantly before and after intervention, and there were also no significant inter-group differences, indicating that PVA particles were safe for the patients.

Furthermore, declined quality of life not only weakens physical function, but also negatively affects social, emotional and mental functions.^24^ To this end, PVA particles are especially suitable for improving the quality of life because fibrous tissues are able to quickly grow into these evenly diametered particles. Notably, large-sized PVA particles may, instead of entirely occupying the vascular lumen, block blood vessels by triggering thrombosis and thus make vascular recanalization possible owing to embolus dislocation.^25^ The two groups scored similarly in physical function, role-physical, bodily pain and general health status before intervention, but the treatment group had significantly higher scores than the control group did six months after intervention (P<0.05).

In summary, interventional embolization therapy can well treat UFs while only mildly affecting ovarian function. Small-sized PVA particles can improve the quality of life by shrinking the uterus and UFs as well as by lowering the risks of complications. Given insufficient fund and long-term observation, only small sample size was used in this study. Meanwhile, quantification of decrease in UF volume, rather than evaluation on the degree of embolization, was conducted herein. Furthermore, changes in ovarian sex hormones were not subjected to long-term follow-up.
